# Differences in COPD Patient Care by Primary Family Caregivers: An Age-Based Study

**DOI:** 10.1371/journal.pone.0107870

**Published:** 2014-09-24

**Authors:** Peng-Ching Hsiao, Chi-Ming Chu, Pei-Yi Sung, Wann-Cherng Perng, Kwua-Yun Wang

**Affiliations:** 1 Graduate Institute of Medical Sciences, National Defense Medical Center, Department of Nursing, Tri-Service General Hospital, Taipei, Taiwan; 2 Section of Health Informatics, Institute of Public Health, National Defense Medical Center and University, Taipei, Taiwan; 3 Department of Nursing, Taoyuan Branch, Taipei Veterans General Hospital, Taoyuan, Taiwan; 4 Division of Pulmonary and Critical Care Medicine, Department of Internal Medicine, Tri-Service General Hospital, National Defense Medical Center, Taipei, Taiwan; 5 Graduate Institute of Medical Sciences, National Defense Medical Center, Department of Nursing, Taipei Veterans General Hospital, Taipei, Taiwan; Federal University of Rio de Janeiro, Brazil

## Abstract

**Background:**

Because Taiwan has the fastest aging rate among developed countries, care for the elderly is becoming more prominent in the country. Primary family caregivers play an important role in patient health and health promotion behavior. Chronic obstructive pulmonary disease (COPD), an age-related disease, is a major public health problem with high morbidity and mortality and can be a long-term burden for family members; however, little attention has been given to the differences in COPD care between elder caregivers and other caregivers. This study aimed to investigate the differences between elder family caregivers and non-elder family caregivers caring for COPD patients in Taiwan, including caring behavior, caregiver response, and caring knowledge.

**Methods:**

This cross-sectional study was conducted between March 2007 and January 2008; 406 primary family caregivers of COPD patients from the thoracic outpatient departments of 6 hospitals in north-central Taiwan were recruited to answer questionnaires measuring COPD characteristics, care behavior, caregiver response, and COPD knowledge. All questionnaires, which addressed caregiver knowledge, care behaviors, and care reactions, were shown to have acceptable validity and reliability, and the data were analyzed using univariate and generalized linear model techniques.

**Results:**

The elder caregivers group had 79 participants, and the non-elder caregivers comprised 327 participants. The COPD-related knowledge scale results were positively correlated with the family caregiver caring behavior scale, suggesting that better COPD-related knowledge among family caregivers may result in improved caring behavior. After adjusting for all possible confounding factors, the elder caregivers had significantly lower COPD-related knowledge than the non-elder caregivers (P<0.001). However, there were no significant differences in the family caregiver caring behavior scale or the caregiver reaction assessment scale between the two groups.

**Conclusions:**

Elder family caregivers require increased education regarding medications and preventive care in COPD patient care.

## Introduction

The aging of the population is a global phenomenon that is both inevitable and predictable [1]. There is no exception in Taiwan. The proportion of Taiwanese elders aged over 65 reached 10.7 percent in 2011 and is expected to be 14 percent in 2017 and 20 percent in 2025. The Taiwanese old-age dependency ratio (((age>65)/(age 15–64))*100) was 7∶1 in 2011 and is expected to be 4∶1 in 2022 and 2∶1 in 2039. Taiwan will be a super-aged society, and its aging rate will be the fastest among developed countries [2]. Thus, elder care by elders will become more common. However, with the population quickly aging and productivity rapidly declining, people in Taiwan will assume larger medical expenses and greater costs for long-term care [2]. Family caregiving represents the first and predominant source of care for 75 to 80% of people with chronic illnesses. Family can be an entity responsible for the care of a sick person [3]. Primary caregivers, especially family caregivers, play an important role in patient health and health promotion behavior [4]–[7]. Caring for patients with chronic disease often exhausts caregivers and has a negative psychological impact on caregivers [8]. In people with chronic illnesses, increased knowledge can reduce the negative psychological impact on the patients or caregivers, including anxiety and depression, and can enhance care efficiency [9]–[11]. Therefore, knowledge may maximize the quality of caring behavior and decrease the potential negative effects on caregivers. Caregivers with negative caring behavior, overloaded caregiver burdens, and a lack of caring knowledge lead to negative patient health and health promotion behavior outcomes [6], [12]–[14]. Therefore, the caregivers' caring behavior, caregiver burden, and caring knowledge may inevitably affect the patients. However, little is known concerning COPD-related knowledge, caring behavior, and the burden on primary family caregivers in Taiwan. The elderly not only have higher physical and mental burdens than other age groups, but they also have higher economic burdens [15]–[17]. Little is known about elder family members as primary caregivers [12], especially regarding the difference between elder primary family caregivers and non-elder primary family caregivers. We hypothesized that elder primary family caregivers have different caring behavior, higher caregiver burdens, and less caring knowledge than young primary family caregivers due to the elders' physical and psychosocial development.

Chronic obstructive pulmonary disease (COPD) is a major public health problem that affects approximately 1/3 of adults and 1/5 of subjects above 65 years old; the prevalence of COPD is high and increasing [18]. Aging is associated with a marked increase in the prevalence of COPD [18], [19]. Furthermore, the majority of epidemiological studies on COPD, which is usually identified as an age-related disease, have focused on the elderly population [20], [21]. Additionally, respiratory symptoms, hospitalization, morbidity, mortality, depression, and anxiety have been reported to be higher in subjects with a diagnosis of COPD than in patients with other respiratory diseases [18], [20], [21]. As a result, COPD can become a long-term burden for family members who serve as day-to-day caregivers and may cause healthcare systems to incur substantial costs [22]. Elder caregivers who care for COPD patients may have higher caregiver burdens than other caregivers. This study aimed to investigate the differences between elder family caregivers and non-elder family caregivers in caring for COPD patients, including caring behavior, caregiver response, and caring knowledge. The results of this study may assist health practitioners in designing appropriate interventions to improve elder caregiver burden and allow elders to age actively.

## Methods

### Participants

This cross-sectional study was conducted between March 2007 and January 2008. Four hundred and six primary family caregivers were recruited from the thoracic outpatient departments of 3 medical centers and 3 regional hospitals in north-central Taiwan using convenience sampling. Primary family caregivers were defined as family members who provided regular, nearly daily care for a family member diagnosed with COPD as defined by the Global Initiative for Chronic Obstructive Lung Disease (GOLD) [23]. The inclusion criteria were being a primary family caregiver of a COPD patient, having normal cognitive functioning (obtaining SPMSQ scores of 8 or above) [24], and self-reporting the absence of psychiatric illness, such as anxiety, depression, bipolar, schizophrenia and dementia. Participants aged 65 years or older were classified as elder caregivers, and participants below 65 years old were classified as non-elder caregivers. A pilot study was conducted one month before the formal study. We collected data from 20 participants for the pilot study. All participants were individually administered the questionnaires in an office near the outpatient department. The participants were asked to respond to the questions either by writing their answers or by responding orally to the investigator and immediately returned the completed questionnaires on site. The participant response rate was 100 percent.

### Ethics

This study was approved by the Institutional Review Board in Taiwan (IRB NO. TSGHIRB 096-05-015). Written informed consent was obtained from all participants.

### Measuring instruments

Data were collected by administering 4 questionnaires, including one questionnaire on the participants' background information, the family caregiver caring behavior scale (FCCBS), the caregiver reaction assessment scale (CRAS), and the COPD-related knowledge scale (CRKS).

In terms of the participants' background information, the following data were collected: age, sex, marital status, religious status, highest educational level, socioeconomic status, sharing responsibility with family (the caregiver could share caregiving during with other family members), presence of chronic disease, residence with the patient, relationship to the patient, presence of other care-needed person(s) at home, and frequency of care.

The family caregiver caring behavior scale (FCCBS) was revised from the Family Caregivers of Cancer Patients Care-Taking Scale [25]. The scale was developed in Chinese. The scale is composed of 49 questions and divided into 4 subscales: care of companionship and monitoring, alternative care of social and general affairs, care of communication and emotion, and care of maintaining physical function. Zero points indicate no need to execute, 1 point indicates does not really need to execute, 2 points indicate sometimes needs to execute, 3 points indicate usually needs to execute, and 4 points indicate always need to execute. A higher score indicates relatively more caring behavior from the primary family caregiver. The professional validity of this score was assessed by the content validity index (CVI) [26], which was 0.98. The internal consistency was evaluated by Cronbach's α coefficient; this coefficient was 0.76 for the total scale and 0.30-0.70 for the subscales in the pilot study and 0.96 for the total scale and 0.88–0.93 for the subscales in the full study.

The caregiver reaction assessment scale (CRAS) developed by Given et al. in 1992 is used to evaluate a caregiver's perceptions of caregiving and reflects the actual workload of a caregiver [27]. We used the Chinese translation of the CRAS. The scale is composed of 24 questions. A 5-point Likert score is used, with higher scores indicating a relatively more positive reaction by the primary family caregiver and a lower actual workload. The professional validity of this score was assessed by the CVI [26], which was 1.00; the internal consistency was evaluated by Cronbach's α coefficient, which was 0.82 for the total scale in the pilot study and 0.62 for the total scale in the full study.

The COPD-related knowledge scale (CRKS) was a modified version of the Bristol COPD Knowledge Questionnaire (BCKQ), which was originally developed by White et al. in 2006 to evaluate changes in COPD patient knowledge after education [28]. In this study, we used the CRKS to represent the primary family caregivers' knowledge level about COPD. After translation and back translation, experts reviewed the scale question by question for appropriateness. According to the experts' suggestions, we added a question regarding nutrition issues with COPD and used 8 parts of the original scale, including understanding of COPD, elimination of sputum, inhaled bronchodilators, inhaled steroids, antibiotic therapy, exercise, smoking cessation, and vaccines. Each part of the scale contained 5 questions. One point was given if the primary family caregiver responded with a correct answer. In contrast, no point was given if the primary family caregiver responded with an incorrect answer or responded that he/she did not know the correct answer. A higher score indicated a higher level of COPD-related knowledge. The professional validity of this score was assessed by the CVI [26], which was 0.98; the internal consistency was evaluated by Kuder-Richardson Formula 20 and was 0.76.

### Statistical analyses

Continuous and categorical variables are presented as the means ± SD and the numbers (percentage), respectively. In the univariate analysis, independent *t*-tests and one-way analysis of variance (ANOVA) were performed to examine differences between the categorical variables and the COPD-related knowledge scale (CRKS); the Mann-Whitney U test was performed to examine differences between the categorical variables and the family caregiver caring behavior scale (FCCBS) and the caregiver reaction assessment scale (CRAS). Spearman's rank correlation was implemented to analyze the correlation between continuous variables, as implemented by Garrod et al. [29]. Factors with P values≤0.05 in the univariate analysis were included in a generalized linear model (GLM) to control for their effects and determine the differences in the FCCBS, caregiver response scale, and CRKS between the elder family caregivers and the non-elder family caregivers. Cronbach's α coefficient was used to determine the reliability, and the CVI was used to validate the scales. All 2-sided statistics were performed with SPSS 17.0 statistical software (SPSS, Chicago, Illinois). Statistical significance was defined as a P value<0.05.

## Results

Table 1 summarizes the characteristics of all 406 primary family caregivers of COPD patients. The elder caregivers group had 79 participants, and the non-elder caregivers group had 327 participants. Almost 1/4 of the primary family caregivers were elders. Both groups had more females than males. The majority of both groups was married, was religious, had low socioeconomic status, and was living with the COPD patient. In both groups, few participants reported having other care-needed person(s) at home. There were no significant differences between the two groups regarding being religious or weekly days of care. However, there were significant differences between the two groups in other background characteristics. The elder group had more females than the non-elder group. More than half of the elder caregivers were the spouses of the patients, with an elementary school education level or below; the elder caregivers had been caring for the patient for a mean of 7.3 years, 6.4 days per week, and 17.9 hours per day. Among the non-elder caregivers, more than half were the son or daughter of the patient, with an education level above senior high school; the non-elder caregivers had been caring for the patient for a mean of 5.2 years, 6.1 days per week, and 12.6 hours per day. The elder group had lower socioeconomic status and more daily hours and duration of care than the non-elder group. A higher proportion of the elder group was living with the COPD patient and had other care-needed person(s) at home compared with the non-elder group.

Three-quarters of the elder caregivers could not share caregiving responsibilities with family at home on a daily basis. However, half of the non-elder caregivers could share caregiving duties with family at home on a daily basis. Almost half of the elder group reported having a chronic illness themselves, whereas only 22.9 percent of the non-elder group reported having a chronic illness themselves.

**Table 1:** Characteristics of study participants.

Characteristic	Elder caregivers (n = 79)	Non elder caregivers (n = 327)	*P*
**Sex, no. (%)**			0.002*
Male	17(21.5)	131(40.1)	
Female	62(78.5)	196(59.9)	
**Married, no. (%)** #	73(92.4)	271(82.9)	0.035*
**Religious, no. (%)**	68(86.1)	254(77.7)	0.098
**Highest educational level, no. (%)**			<0.001*
Elementary school	55(69.6)	69(21.1)	
Junior high school	8(10.1)	50(15.3)	
Senior high school	7(8.9)	100(30.6)	
College or above	9(11.4)	107(32.7)	
**Social economic status, no. (%)**			<0.001*
Low	67(84.8)	151(46.2)	
Moderate	5(6.3)	85(26.0)	
High	7(8.9)	91(27.8)	
**Sharing responsibility with family, no. (%)**	20(25.3)	185(56.6)	<0.001*
**Chronic disease, no. (%)**	39(49.4)	75(22.9)	<0.001*
**Living with patient, no. (%)**	72(91.1)	243(74.3)	0.001
**Relation with patient**			<0.001*
Spouse	66(83.5)	95(29.1)	
Son or daughter	79(8.9)	201(61.5)	
others	6(7.6)	31(9.5)	
**Other care-needed person(s) in home, no. (%)**	3(3.8)	49(15.0)	0.008*
**Duration of care, mean± SD, y**	7.3±6.0	5.2±3.9	<0.001*
**Weekly days of care, mean ± SD**	6.4±1.8	6.1±2.0	0.014*
**Daily hours of care, mean ± SD**	17.9±8.3	12.6±8.7	0.353

* The *P* value≦0.05.

# Widow, widower, separated couple, or divorced people were not included.

Table 2 shows the results of the Spearman rank correlation between the caregivers' total COPD-related knowledge scale (CRKS) scores, the family caregiver caring behavior scale (FCCBS) scores, the caregiver reaction assessment scale (CRAS) scores, and the continuous characteristics. The CRKS scores were negatively correlated with daily hours of care, suggesting that high COPD-related knowledge may result in more effective daily care. The FCCBS scores were positively correlated with daily hours of care and negatively correlated with weekly days of care. Moreover, the CRAS scores were negatively correlated with daily hours of care, suggesting that more caring behavior may result in increased daily hours of care and, thus, more negative reactions and fewer weekly days of care.

**Table 2:** Correlation between CRKS, FCCBS, CRAS, and continuous characteristics.

Variable	FCCBS (r (*P*))	CRAS (r (*P*))	CRKS (r (*P*))
**Duration of care**	−0.03(0.576)	−0.01(0.867)	0.03(0.603)
**Weekly days of care**	−0.11(0.033)*	0.02(0.725)	0.09(0.083)
**Daily hours of care**	0.13(0.011)*	−0.16(0.001)*	−0.15(0.002)*

* The *P* value≦0.05.

FCCBS: Family Caregiver Caring Behaviors scale; CRAS: Caregiver Reaction Assessment scale; CRKS: COPD-related Knowledge scale.

Table 3 presents the comparison of the caregivers' total COPD-related knowledge scale (CRKS) scores, the family caregiver caring behavior scale (FCCBS), and the caregiver reaction assessment scale (CRAS) among family caregivers with different categorical characteristics. The results indicate that differences in the caregivers' marital status, the presence of other care-needing person(s) at home, and the caregivers' relationship to the patient were related to the caregivers' FCCBS scores. There were significant differences in CRAS scores between male and female caregivers, and among caregivers with different highest educational levels, socioeconomic status, rotations of responsibility with family, other (chronic) disease, and other care-needed person(s) at home. In addition, differences in the caregivers' sex, marital status, educational level, socioeconomic status, rotation of responsibility with family, and relationship to the patient were related to the caregivers' CRKS scores.

**Table 3:** Comparison of CRKS, FCCBS, CRAS between different categorical characteristics.

Characteristic	FCCBS mean ± SD	*P* #	CRAS mean ± SD	*P* #	CRKS mean ± SD	*P* §
**Age**		0.563		0.032*		<0.001
Elder	97.8±33.8		74.1±6.9		21.7±4.1	
Non elder	101.9±37.6		76.0±9.1		24.8±5.5	
**Sex**		0.078		<0.001*		0.002*
Male	96.4±34.3		77.6±8.3		25.3±5.4	
Female	103.9±38.2		74.5±8.8		23.6±5.3	
**Marriage**		0.023*		0.745		0.014*
Single	114.2±45.8		75.0±10.6		23.8±6.1	
Married	98.8±34.6		75.8±8.4		24.3±5.3	
**Religion**		0.110		0.702		0.810
No	105.3±36.2		76.0±10.2		24.1±5.1	
Yes	100.1±37.1		75.6±8.3		24.2±5.5	
**Highest educational level**		0.267		<0.001*		<0.001*
Elementary school	96.0±33.0		74.3±7.9		21.9±4.8	
Junior high school	100.5±39.0		75.4±7.2		23.0±5.2	
Senior high school	107.3±40.3		74.5±8.7		24.6±5.0	
College or above	100.9±36.1		78.2±9.9		26.8±5.2	
**Low social economic status**		0.956		0.002*		<0.001*
No	101.2±37.9		76.9±9.5		26.1±5.2	
Yes	101.1±36.1		74.6±7.9		22.6±5.0	
**Sharing responsibility with family**		0.054		0.017*		<0.001*
No	97.5±34.6		74.8±8.2		22.8±5.2	
Yes	104.7±38.8		76.5±9.2		25.6±5.2	
**Chronic disease**		0.508		<0.001*		0.120
No	101.9±37.3		76.7±8.4		24.5±5.5	
Yes	99.1±36.1		73.0±9.2		23.5±5.0	
**Living with patient**		0.457		0.135		0.932
No	101.3±33.3		77.0±8.5		24.2±5.5	
Yes	101.1±38.0		75.3±8.8		24.2±5.3	
**Other care-needed person(s) in home**		<0.001*		0.001*		0.471
No	97.8±35.0		76.2±8.5		24.1±5.3	
Yes	123.9±41.6		71.6±9.4		24.7±5.5	
**Spouse is main caregiver**		0.022*		0.194		<0.001*
No	104.5±38.2		76.0±9.1		25.1±5.7	
Yes	96.0±34.4		75.2±8.1		22.8±4.5	

*P* **#** The *P* values were derived from Mann-Whitney U test.

*P* **§** The *P* values were derived from independent *t*-test or ANOVA.

* The *P* value≦0.05.

FCCBS: Family Caregiver Caring Behaviors scale; CRAS: Caregiver Reaction Assessment scale; CRKS: COPD-related Knowledge scale.

Table 4, Table 5 and Table 6 show the results of a GLM controlling for confounding effects to determine differences in family caregiver caring behaviors, caregiver responses, and COPD-related knowledge between the elder family caregivers and the non-elder family caregivers. Variables that significantly correlated with the family caregiver caring behaviors scale, caregiver response scale, and COPD-related knowledge scale (CRKS) from the univariate analysis and significant subject characteristics (P<0.05) were adjusted for use in the GLM. The results of the GLM showed that after adjusting for the correlated variables, the elder group had significantly lower COPD-related knowledge scores than the non-elder group. However, there were no significant differences between the two groups in family caregiver caring behaviors scores or caregiver response scores. Furthermore, we analyzed differences in the subscales of the CRKS between the two groups using the GLM. The results indicated that after adjusting for the correlated variables, the elder group had significantly lower scores than the non-elder group in the understanding of COPD, inhaled bronchodilators, inhaled steroids, antibiotic therapy, smoking cessation, and vaccines (Table 5 and Table 6).

**Table 4:** Generalized linear model to analyze the difference of FCCBS, subscale of FCCBS, and CRAS in different factors.

Factors	FCCBS		Care of companionship and monitoring		Alternative care of social and general affairs		Care of communication and emotion		Care of maintaining physical function		CRAS	
	β(95%CI)	*P*	β(95%CI)	*P*	β(95%CI)	*P*	β(95%CI)	*P*	β(95%CI)	*P*	β(95%CI)	*P*
**Elder vs. non elder caregivers**	−4.0(−13.0∼5.1)	0.388	−1.1(−4.8∼2.5)	0.541	−1.8(−4.7∼1.2)	0.242	−0.9(−3.4∼1.6)	0.476	−0.2(−1.1∼0.8)	0.743	−1.9(−4.1∼0.2)	0.079
**Sex** (Female vs. male)	7.3(−0.1∼14.7)	0.053	2.2(−0.8∼5.2)	0.153	2.9(0.5∼5.4)	0.017*	2.1(0.0∼4.2)	0.047*	0.1(−0.7∼0.9)	0.822	−3.2(−4.9∼−1.4)	<0.001*
**Marriage** (Married vs. single)	−15.6(−25.4∼−5.7)	0.002*	−6.0(−10.0∼−2.0)	0.003*	−4.7(−7.9∼−1.5)	0.004*	−2.5(−5.3∼0.2)	0.071	−2.3(−3.3∼−1.3)	<0.001*	0.8(−1.6∼3.1)	0.525
**Highest educational level**												
Elementary school vs. college or above	−4.9(−14.1∼4.4)	0.305	−1.1(−4.9∼2.6)	0.553	−2.0(−5.0∼1.1)	0.205	−1.4(−4.0∼1.2)	0.288	−0.3(−1.3∼0.6)	0.483	−3.8(−6.0∼−1.7)	0.001*
Junior high school vs. college or above	−0.4(−12.0∼11.1)	0.943	0.9(−3.8∼5.6)	0.705	−0.4(−4.2∼3.3)	0.823	−0.6(−3.8∼2.6)	0.721	−0.3(−1.5∼0.9)	0.614	−2.8(−5.5∼−0.1)	0.042*
Senior high school vs. college or above	6.4(−3.2∼16.0)	0.192	1.8(−1.1∼6.7)	0.166	2.3(−0.9∼5.4)	0.154	0.6(−2.1∼3.3)	0.664	−0.8(−0.2∼1.8)	0.137	−3.6(−5.9∼−1.4)	0.002*
**Low social economic status** (Yes vs. no)	0.1(−7.1∼7.3)	0.969	−0.3(−3.2∼2.7)	0.867	0.1(−2.3∼2.4)	0.947	0.5(−1.5∼2.5)	0.593	−0.2(−1.0∼0.5)	0.544	−2.3(−4.0∼−0.6)	0.008*
**Sharing responsibility with family** (Yes vs. no)	7.0(−0.1∼14.1)	0.055	3.9(1.0∼6.8)	0.008*	1.8(−0.5∼4.2)	0.130	0.2(−1.7∼2.2)	0.809	1.0(0.3∼1.8)	0.008*	1.2(0.0∼3.4)	0.055
**Chronic disease** (Yes vs. no)	−2.7(−10.6∼5.3)	0.513	−1.2(−4.5∼2.0)	0.460	−0.8(−3.4∼1.8)	0.548	0.1(−2.2∼2.3)	0.965	−0.7(−1.5∼0.1)	0.103	−3.7(−5.5∼−1.8)	<0.001*
**Other care-needed person(s) in home** (Yes vs. no)	26.2(15.8∼36.7)	<0.001*	7.1(2.9∼11.4)	0.001*	10.2(6.8∼13.6)	<0.001*	7.1(4.2∼10.0)	<0.001*	1.8(0.7∼2.9)	0.001*	−4.6(−7.1∼−2.1)	<0.001*
**Spouse is main caregiver** (Yes vs. no)	−8.3(−15.6∼−1.0)	0.026*	−4(−6.9∼−1.0)	0.008*	−2.9(−5.2∼−0.5)	0.019*	−0.5(−2.6∼1.5)	0.618	−0.9(−1.7∼−0.2)	0.017*	−0.8(−2.5∼1.0)	0.391

*The *P* value≦0.05.

FCCBS: Family Caregiver Caring Behaviors scale; CRAS: Caregiver Reaction Assessment scale.

Adjusted variable: Sex, Marriage, Highest Educational Level, Social Economic Status, Rotary, Other (chronic) disease, Living with patient, Other care-needed person(s) in home, Spouse is the main caregiver, Duration of care, Daily hours of care.

**Table 5:** Generalized linear model to analyze the difference of CRKS, and subscale of CRKS in different factors.

Factors	CRKS		Understanding of COPD		Elimination of sputum		Inhaled bronchodilator		Inhaled steroids	
	β(95%CI)	*P*	β(95%CI)	*P*	β(95%CI)	*P*	β(95%CI)	*P*	β(95%CI)	*P*
**Elder vs. non elder caregivers**	−3.1(−4.3∼−1.8)	<0.001*	−0.7(−1.0∼−0.4)	<0.001*	−0.2(−0.5∼0.1)	0.232	−0.5(−0.7∼−0.2)	0.002*	−0.4(−0.6∼−0.1)	0.001*
**Sex** (Female vs. Male)	−1.7(−2.8∼−0.6)	0.002*	−0.3(−0.6∼−0.1)	0.012*	−0.1(−0.3∼0.2)	0.606	−0.3(−0.5∼−0.1)	0.007*	−0.2(−0.4∼0)	0.044*
**Marriage** (Married vs. Single)	0.5(−1∼1.9)	0.542	−0.1(−0.4∼0.2)	0.574	0.2(−0.1∼0.5)	0.248	0.1(−0.2∼0.4)	0.677	−0.1(−0.3∼0.2)	0.625
**Highest educational level**										
Elementary school vs. college or above	−4.9(−6.2∼−3.6)	<0.001*	−0.8(−1.1∼−0.5)	<0.001*	−0.5(−0.8∼−0.2)	0.001*	−0.5(−0.7∼−0.2)	0.002*	−0.4(−0.6∼−0.1)	0.002*
Junior high school vs. college or above	−3.8(−5.4∼−2.2)	<0.001*	−0.6(−1.0∼−0.3)	0.001*	−0.4(−0.8∼−0.1)	0.016*	−0.2(−0.6∼0.2)	0.260	−0.4(−0.7∼−0.1)	0.003*
Senior high school vs. college or above	−2.2(−3.5∼−0.9)	0.001	−0.3(−0.6∼0.1)	0.123	−0.3(−0.6∼0)	0.034*	−0.19−0.4∼0.2)	0.525	0(−0.2∼0.2)	0.985
**Low social economic status** (Yes vs. no)	−3.5(−4.5∼−2.5)	<0.001*	−0.6(−0.9∼−0.4)	<0.001*	−0.3(−0.5∼−0.1)	0.017*	−0.4(−0.6∼−0.1)	0.001*	−0.4(−0.6∼−0.3)	<0.001*
**Sharing responsibility with family** (Yes vs. no)	2.8(1.8∼3.9)	<0.001*	0.4(0.2∼0.6)	0.001*	0.4(0.2∼0.7)	<0.001*	0.5(0.3!0.7)	<0.001*	0.2(−0.1∼0.3)	0.097
**Chronic disease** (Yes vs. no)	−0.9(−2.1∼0.2)	0.123	−0.2(−0.4∼0.1)	0.205	0(−0.2∼0.3)	0.734	−0.2(−0.5∼0)	0.090	−0.1(−0.3∼0.1)	0.510
**Other care-needed person(s) in home** (Yes vs. no)	0.6(−1.0∼2.2)	0.462	0.1(−0.2∼0.5)	0.514	0.1(−0.2∼0.4)	0.573	0(−0.4∼0.3)	0.876	−0.1(−0.3∼0.2)	0.540
**Spouse is main caregiver** (Yes vs. no)	−2.3(−3.4∼−1.3)	<0.001*	−0.4(−0.6∼−0.1)	0.002*	−0.1(−0.3∼0.2)	0.586	−0.4(−0.7∼−0.2)	<0.001*	−0.2(−0.4∼−0.1)	0.008*

* The *P* value≦0.05.

CRKS: COPD-related Knowledge scale.

Adjusted variable: Sex, Marriage, Highest Educational Level, Social Economic Status, Rotary, Other (chronic) disease, Living with patient, Other care-needed person(s) in home, Spouse is the main caregiver, Duration of care, Weekly days of care, Daily hours of care.

**Table 6:** Generalized linear model to analyze the difference of CRKS, and subscale of CRKS in different factors.

Factors	Antibiotics therapy		Exercise		Quit smoking		Nutrition		Vaccine	
	β(95%CI)	*P*	β(95%CI)	*P*	β(95%CI)	*P*	β(95%CI)	*P*	β(95%CI)	*P*
**Elder vs. non elder caregivers**	−0.9(−1.2∼−0.5)	<0.001*	−0.1(−0.3∼0.1)	0.593	−0.2(−0.4∼0)	0.014*	−.1(−0.1∼0.4)	0.300	−0.4(−0.6∼−0.1)	0.005*
**Sex** (Female vs. male)	−0.5(−0.8∼−0.2)	0.001*	−0.1(-0.2∼0.1)	0.533	−0.2(−0.3∼0)	0.022*	−0.1(−0.3∼0.1)	0.565	0(−0.3∼0.2)	0.721
**Marriage** (Married vs. single)	−0.3(−0.7∼0.1)	0.140	0.2(0∼0.4)	0.079	−0.1(−0.3∼0.1)	0.430	0.5(0.3∼0.8)	<0.001*	0(−0.3∼0.3)	0.891
**Highest educational level**										
Elementary school vs. college or above	−1.4(−1.7∼−1.1)	<0.001*	−0.2(−0.4∼0)	0.027*	−0.4(−0.6∼−0.2)	<0.001*	0(−0.2∼0.3)	0.842	−0.7(−1.0∼−0.5)	<0.001*
Junior high school vs. college or above	−1.0(−1.4∼−0.6)	<0.001*	−0.1(−0.3∼0.1)	0.589	−0.3(−0.5∼−0.1)	0.008*	0(−0.4∼0.3)	0.836	−0.6(−0.9∼−0.3)	<0.001*
Senior high school vs. college or above	−0.8(−1.1∼−0.4)	<0.001*	−0.1(−0.3∼0.10	0.571	−0.1(−0.3∼0.1)	0.217	−0.1(−0.3∼0.2)	0.578	−0.4(−0.7∼−0.2)	0.001*
**Low social economic status** (Yes vs. no)	−0.8(−1.1∼−0.5)	<0.001*	−0.1(−0.3∼0)	0.114	−0.3(−0.5∼−0.2)	<0.001*	−0.1(−0.3∼0.1)	0.559	−0.4(−0.6∼−0.2)	<0.001*
**Sharing responsibility with family** (Yes vs. no)	0.7(0.4∼1.0)	<0.001*	0.1(−0.1∼0.2)	0.361	0.1(0∼0.3)	0.082	0(−0.2∼0.2)	0.840	0.4(0.2∼0.6)	<0.001*
**Chronic disease** (Yes vs. no)	−0.3(−0.7∼0)	0.031*	−0.1(−0.3∼0.1)	0.291	−0.1(−0.2∼0.1)	0.292	0.1(−0.2∼0.3)	0.660	0(−0.3∼0.2)	0.707
**Other care**−**needed person(s) in home** (Yes vs. no)	0.3(−0.2∼0.7)	0.243	0.2(0∼0.5)	0.056	0(−0.2∼0.2)	0.890	−0.2(−0.5∼0.1)	0.168	0.2(−0.1∼0.5)	0.240
**Spouse is main caregiver** (Yes vs. no)	−0.7(−1.0∼−0.5)	<0.001*	0(−0.2∼0.2)	0.980	−0.2(−0.4∼−0.1)	0.002*	0.2(0∼0.4)	0.119	−0.4(−0.6∼−0.2)	0.001*

*The *P* value≦0.05.

Adjusted variable: Sex, Marriage, Highest Educational Level, Social Economic Status, Rotary, Other (chronic) disease, Living with patient, Other care-needed person(s) in home, Spouse is the main caregiver, Duration of care, Weekly days of care, Daily hours of care.

## Discussion

In our study, most caregivers were female. With respect to the two groups, we found that the elder group had more females than the non-elder group. The majority of the elder group was spouses, while the majority of the non-elder group was sons or daughters. Most previous studies have indicated that women are the major primary family caregivers, especially in caring for the elderly or patients with chronic disease [6], [8], [12]. Currently, the number of females working is increasing; therefore, the caregiving duties for families with chronic disease have few gender differences in the non-elder group. In Taiwan, spouses remain the primary family caregiver due to Chinese culture, especially in the elder group [30]–[32]. Our data showed that a higher proportion of elder caregivers had low socioeconomic status compared with non-elder caregivers. A reason for this difference might be that the non-elder group primarily consisted of sons or daughters who were still working and saving money. Therefore, elder caregivers have greater potential physical and economic care stress than non-elder caregivers, especially elder female caregivers in Taiwan. Further policies or interventions to reduce care stress among elder caregivers demand immediate attention.

Our study showed that the higher the number of daily hours of care, the higher the family caregivers' caring behaviors and the more negative the family caregivers' caring reactions. These results are consistent with the results of Alpass et al., who showed that caregivers providing higher levels of care reported a more negative psychological response and poorer mental health. The study also demonstrated that caregivers providing higher levels of care and more care across time had poorer health outcomes. Long-term and heavy daily caregiving may have detrimental effects on health status due to a lack of formal support resources and the strain of multiple roles [12].

There were no significant differences between groups in the total caregiver caring behaviors scale scores, nor were there differences in any of the caregiver caring behavior subscale scores. Our data indicate that the older caregivers had a tendency toward less caregiver caring behaviors. Elders are not as strong physically as non-elders, which may explain why the elders tended to have fewer caregiver caring behaviors than non-elders.

Our data indicate that the more caring knowledge caregivers have, the less daily hours of care they provide and, thus, the more positive caring reaction the caregivers have. Research has shown that caring for patients with chronic disease often makes caregivers feel physically and psychologically exhausted and leads to a more negative psychological impact on caregivers [8]. Friedemann et al. also indicated that patients' functional limitations yielded the strongest predictive coefficients followed by caregiver stress [33]. In people with chronic illnesses, increased knowledge can reduce the negative psychological impact on the patients or caregivers, such as anxiety and depression, and can enhance care efficiency [9]–[11]. Another study demonstrated that education reduces subjective burdens, worry, and displeasure and improves intra-psychic strain, depression and all empowerment measures [32].

Our study showed that the elder caregivers' caring reaction was more negative than that of the non-elder caregivers, although this difference was not statistically significant. Most elders have at least one chronic disease and poor income due to retirement. The prevalence of frailty increases with age and is greater in women [34]. Limpawattana et al. reported that older caregivers with poorer self-reported health status, a longer of duration of care, and a lower self-reported income had higher caregiver burdens, such as stress and depression [8]. Studies have also shown that negative care reactions by caregivers lead to negative impacts, such as caregiver depression, poor quality of care, potentially harmful behavior, and elder abuse [6], [14], [35]. Burdened caregivers have reported less social support, poorer quality of life, and problems with social integration [36]. Improving elder caregivers' social support systems and quality of life through respite care and education might be useful in making their caring reaction more positive. Our study showed that elder caregivers have a poor performance on the COPD-related knowledge scale (CRKS), which might be the result of the working memory becoming weaker with increasing age [37]. Another study also showed that the caregivers' advancing age increased the risks related to functional and cognitive impairments [38]. In addition, health professionals might not have much time to provide the caregivers with detailed information about COPD. A study showed that young men use the internet more than the elderly [39]. Younger caregivers might access COPD-related information on the internet, which might also explain the difference in the scores between older and younger participants. The results of the present study make an important contribution to the literature; elder caregivers showed poor performance on the medication-related subscales. This result might be explained by the fact that most health professionals might stereotype older persons with respect to memory and learning new concepts. Therefore, health professionals might not spend much time educating older persons, especially with respect to medication-related information. In addition, younger caregivers have greater access to medication information on the internet [39]. Elder family caregivers need more education on medications and preventive care in patients with COPD.

This study had several limitations. First, the questionnaires may have response bias due to the nature of self-reporting. Second, the subjects were recruited only from northern Taiwan, which may partially limit the generalizability of our results. However, many of these observations are likely applicable to the broader socio-cultural context of Taiwan and Southeast Asia. Third, the severity of COPD was not considered a confounder when the differences between the two groups in caregiver behavior and caregiver response were analyzed, which may somewhat restrict the interpretation of our results. Fourth, although “sharing responsibilities with family” is one type of “help with caregiving”, we did not include the data regarding whether the primary family caregiver had assistance or other help when providing care to the COPD patient. Future studies are needed to clarify whether “help with caregiving” might affect the outcomes. Regarding possible future studies, we might develop a COPD-related knowledge education project for elder caregivers and examine the program's effects on COPD-related knowledge, care, and caregiver burden for elder caregivers. The application of these results to caregivers in the context of other chronic diseases is unknown. Further studies are needed to explore the differences in care for other age-related chronic diseases by primary family caregivers. Furthermore, research on the care needs of elder primary family caregivers caring for an elder family member with chronic disease is worth pursuing in Taiwan.

## Conclusions

In conclusion, our findings indicate that there were no significant differences in family caregiver caring behavior or caregiver reaction between the elder and the non-elder caregivers. However, the elder caregivers had significantly lower COPD-related knowledge than the non-elder caregivers, especially with regard to their understanding of COPD, inhaled bronchodilators, inhaled steroids, antibiotic therapy, smoking cessation, and vaccines. Elder family caregivers need more education on COPD drugs and COPD symptom prevention.
